# Downregulation of lncRNA DANCR promotes osteogenic differentiation of periodontal ligament stem cells

**DOI:** 10.1186/s12861-019-0206-8

**Published:** 2020-01-14

**Authors:** Zhuo Wang, Yuanliang Huang, Luanjun Tan

**Affiliations:** Department of Stomatology, Shanghai East Hospital Affiliated to Tongji University, No.150 Jimo Rd., Shanghai, 200120 China

**Keywords:** Long non-coding RNA (lncRNA), Differentiation antagonizing non-protein coding RNA (DANCR), Osteogenic differentiation, Periodontal ligament stem cells (PDLSCs)

## Abstract

**Background:**

Long non-coding RNAs (lncRNAs) have been widely known to have an appreciable effect in physiology and pathology. In tooth regeneration, periodontal ligament stem cells (PDLSCs) are regarded as a key effector, whereas, how lncRNA acts in the osteogenic differentiation of PDLSCs have not been completely understood. This study aims to find out the relationship between lncRNA DANCR and the proliferation and osteogenic differentiation of PDLSCs.

**Methods:**

Microarray was used to observe the different expression of lncRNAs in differentiated and undifferentiated PDLSCs. And then osteogenic-related lncRNA, DANCR was screened out. Its effects on proliferation and osteogenic differentiation was explored by constructing an overexpression and inhibition model. qRT-PCR was used to detect the mRNA expression of osteogenesis related genes. MTT assay was performed to assess the effects of DANCR on cell growth curve. To quantify the effects of DANCR on osteogenic differentiation of PDLSCs, ALP staining and alizarin red was performed in basic culture medium and osteogenic medium. Data were statistically processed.

**Results:**

Compared with the undifferentiated PDLSCs, the alizarin red staining level was higher in differentiated PDLSCs. And the expressions of osteogenic differentiation marker genes Runt-related transcription factor 2 (Runx2), osteocalcin (OCN) and bone morphogenetic protein (BMP-2) were significantly increased in the differentiated PDLSCs. Furthermore, we noticed that comparing with control groups, the expression of lncRNA DANCR decreases markedly in osteogenically induced PDLSCs. DANCR promoted proliferation of PDLSCs, as evidenced by cell viability. Further investigation has proven that the downregulation of DANCR shows in the calcium sediment forming, alkaline phosphatase (ALP) activation and some osteogenic-related gene markers’ upregulation including Runx2, OCN and BMP-2, which finally results in the osteogenic differentiation of PDLSCs following the transfection and induction. Conversely, DANCR upregulation was shown to repress the osteogenic differentiation potential of PDLSCs.

**Conclusions:**

The osteogenic differentiation of PDLSCs has proven to related to the down regulation of lncRNA DANCR. And this paper throws light on the effects of DANCR in the process of PDLSCs’ osteogenic differentiation.

## Background

Dental problems caused by dental caries, periodontal disease and tooth injury compromise the oral and general health issues [1]. Periodontal diseases, such as periodontitis, are crucial factors of tooth loss [2]. With the potential for the full recovery of tooth function, regenerative therapy for tooth tissue repair and whole-tooth replacement is currently considered the novel therapeutic methods [3].

The periodontium is composed of periodontal ligament (PDL) tissue, alveolar bone, cementum and gingival tissue [4]. Periodontal ligament is a highly vascularized tissue that lies between the cementum and alveolar bone and plays a pivotal role in tooth maintenance [5]. The PDL tissue is fibrotic and vascularized and it is vital for tooth anchorage, facilitating nutrition delivery, repairing and sensation [6]. It has been demonstrated that cells in periodontal ligament contain a heterogeneous population of cells, including osteoblasts, cementoblasts, osteoblasts, myofibroblasts, nerve cells, endothelial cells, fibroblasts, epithelial cells and periodontal ligament stem cells (PDLSCs) [6]. Among these, PDLSCs have shown potential in osteogenic differentiation for periodontal regeneration [7, 8]. Thus, it becomes significant to find the mechanism of osteogenic differentiation of PDLSCs.

Long non-coding RNAs (lncRNAs), which cannot code protein and are more than 200 nt, play crucial roles in both cell fate determination and disease pathogenesis [9]. Previous study reported that downregulating of lncRNANONHSAT009968 inhibited osteogenic differentiation of human bone mesenchymal stem cells [10]. Xiao et al. demonstrate that lncRNA MALAT1 promotes human aortic valve interstitial cells’ osteoblast differentiation [11].

Long non-coding RNA differentiation antagonizing non-protein coding RNA (LncRNA DANCR) was the first lncRNA demonstrated to have relationship with the suppression of progenitor differentiation [12]. The function of promoting chondrogenic differentiation of human synovium-derived mesenchymal stem cells by lncRNA DANCR has been proven [13]. And Jiang et al. indicated that lncRNA DANCR promotes tumor progression and cancer stemness features in osteosarcoma [14]. However, the function of lncRNA DANCR on the osteogenic differentiation of PDLSCs remains unclear.

We currently focus on the effects of lncRNA DANCR on PDLSCs’ osteogenic differentiation. We showed that lncRNA DANCR was significantly downregulated in osteogenically induced PDLSCs compared to that undifferentiated counterparts. Further examination indicated that the inhibition of DANCR was positively related to the PDLSCs’ osteogenic differentiation following the transfection and induction. However, DANCR upregulation was shown to repress PDLSCs’ osteogenic differentiation. These data suggest that lncRNA DANCR get involved in cell osteogenic differentiation of PDLSCs.

## Results

### LncRNA DANCR was down-regulated in differentiated PDLSCs

**Fig. 1:**
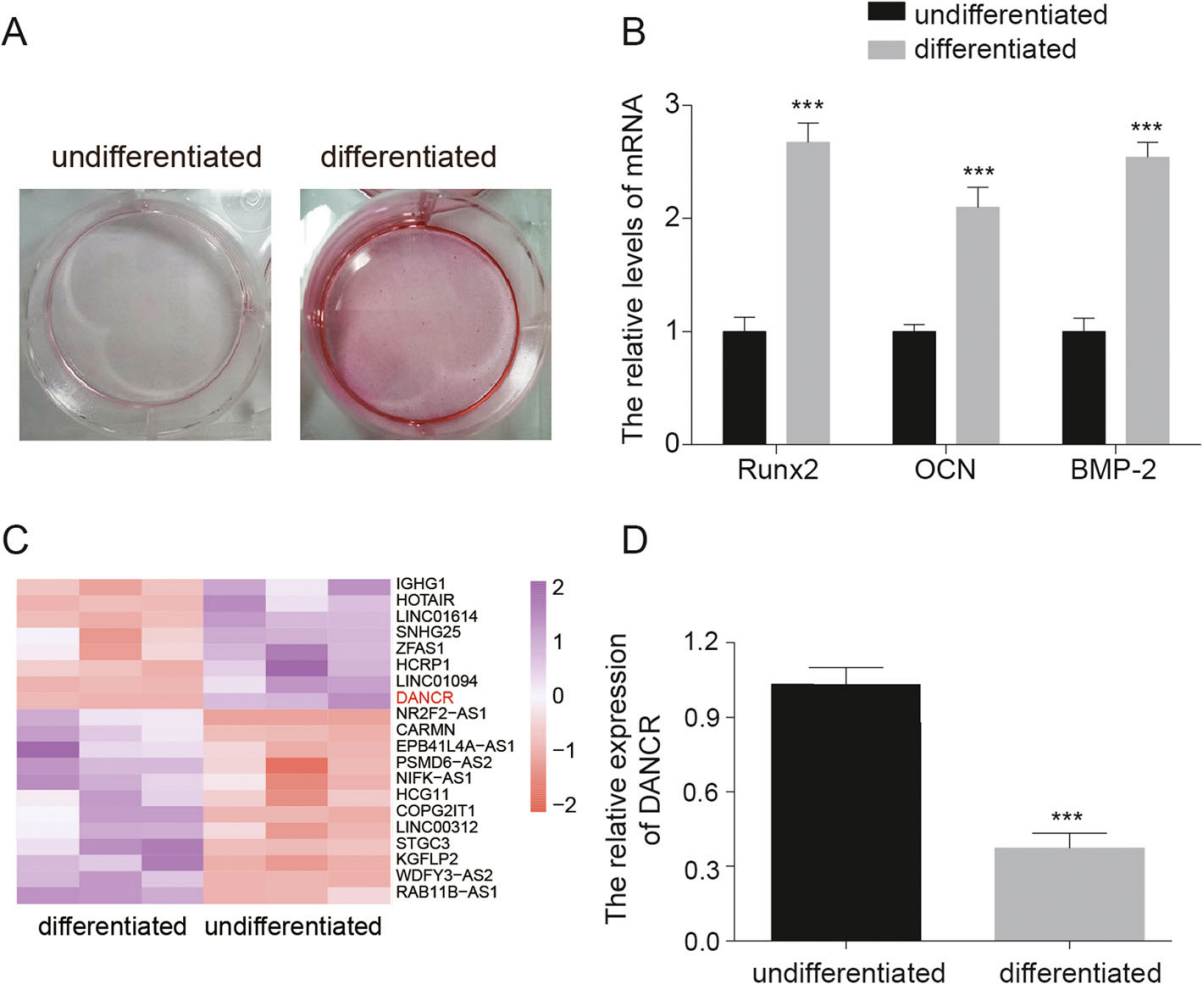
LncRNA DANCR levels was down-regulated in differentiated PDLSCs. **a** Alizarin red staining assay was performed to measure the calcium deposits in PDLSCs and the alizarin red staining level was higher in differentiated PDLSCs compared with that in undifferentiated PDLSCs. **b** The expression of osteogenic differentiation marker genes (including Runx2, OCN and BMP-2) were detected by qRT-PCR, and the result showed that the expressions of Runx2, OCN and BMP-2 were significantly increased in differentiated PDLSCs, ***P < 0.001, compared with undifferentiated PDLSCs group. **c** The differential expressions of lncRNA were identified by microarray in differentiated and undifferentiated PDLSCs. LncRNA DANCR was markedly down-regulated. **d** The result of qRT-PCR showed that the expressions of DANCR was significantly decreased in differentiated PDLSCs, ***P < 0.001, compared with undifferentiated PDLSCs group

The alizarin red staining was performed to examine the osteogenic differentiation of PDLSCs. As showed in Fig. 1a, higher alizarin red staining level was observed in differentiated PDLSCs compared with undifferentiated PDLSCs. The result of qRT-PCR revealed that the expressions of osteogenic differentiation marker genes, including Runx2, OCN and BMP-2, were notably increased in differentiated PDLSCs compared with the undifferentiated PDLSCs (Fig. 1b). These results suggested that PDLSCs were successfully inducted into differentiated PDLSCs. Next, the differential expressions of lncRNA were identified by microarray assay, and the result showed that lncRNA DANCR was significantly downregulated in differentiated PDLSCs (Fig. 1c). To confirm the results obtained from the microarray analysis, we performed a more thorough examination of DANCR expression in undifferentiated and differentiated PDLSCs by qRT-PCR. The result indicated that the expression of lncRNA DANCR was significantly down-regulated in differentiated PDLSCs compared with that in the undifferentiated PDLSCs (Fig. 1d).

### Downregulation of DANCR inhibited the proliferation of PDLSCs

**Fig. 2:**
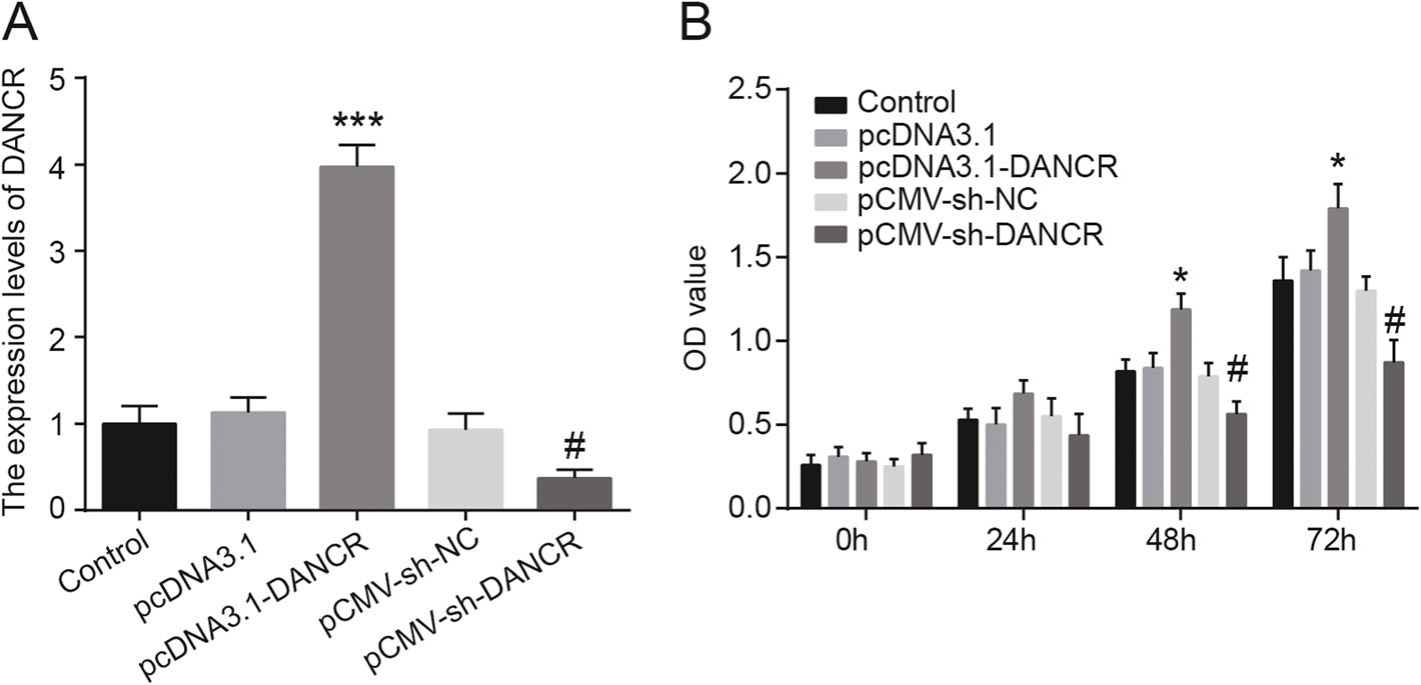
DANCR promotes the proliferation of PDLSCs. **a** The mRNA levels of DANCR were detected by qRT-PCR. The expression of lncRNA DNACR was significantly increased in pcDNA3.1-DANCR group while it was notably decreased in pCMV-sh-DANCR group. **b** Cell viability was measured by MTT assay, the result showed that cell viability was increased in pcDNA3.1-DANCR group while it was decreased in pCMV-sh-DANCR group, *P < 0.05, ***P < 0.001, compared with the pcDNA3.1 group; # *P* < 0.05 compared with the pCMV-sh-NC group

After transfection, the result of qRT-PCR showed that the expression of lncRNA DNACR was significantly increased in pcDNA3.1-DANCR group while it was notably decreased in pCMV-sh-DANCR group (Fig. 2a). The result of MTT assay showed that the cell viability was enhanced in pcDNA3.1-DANCR group while it showed the opposite effects in pCMV-sh-DANCR group (Fig. 2b). These results suggest that DNACR could promote the proliferation of PDLSCs.

### Downregulation of DANCR is positively correlated with the osteogenic differentiation of PDLSCs

**Fig. 3:**
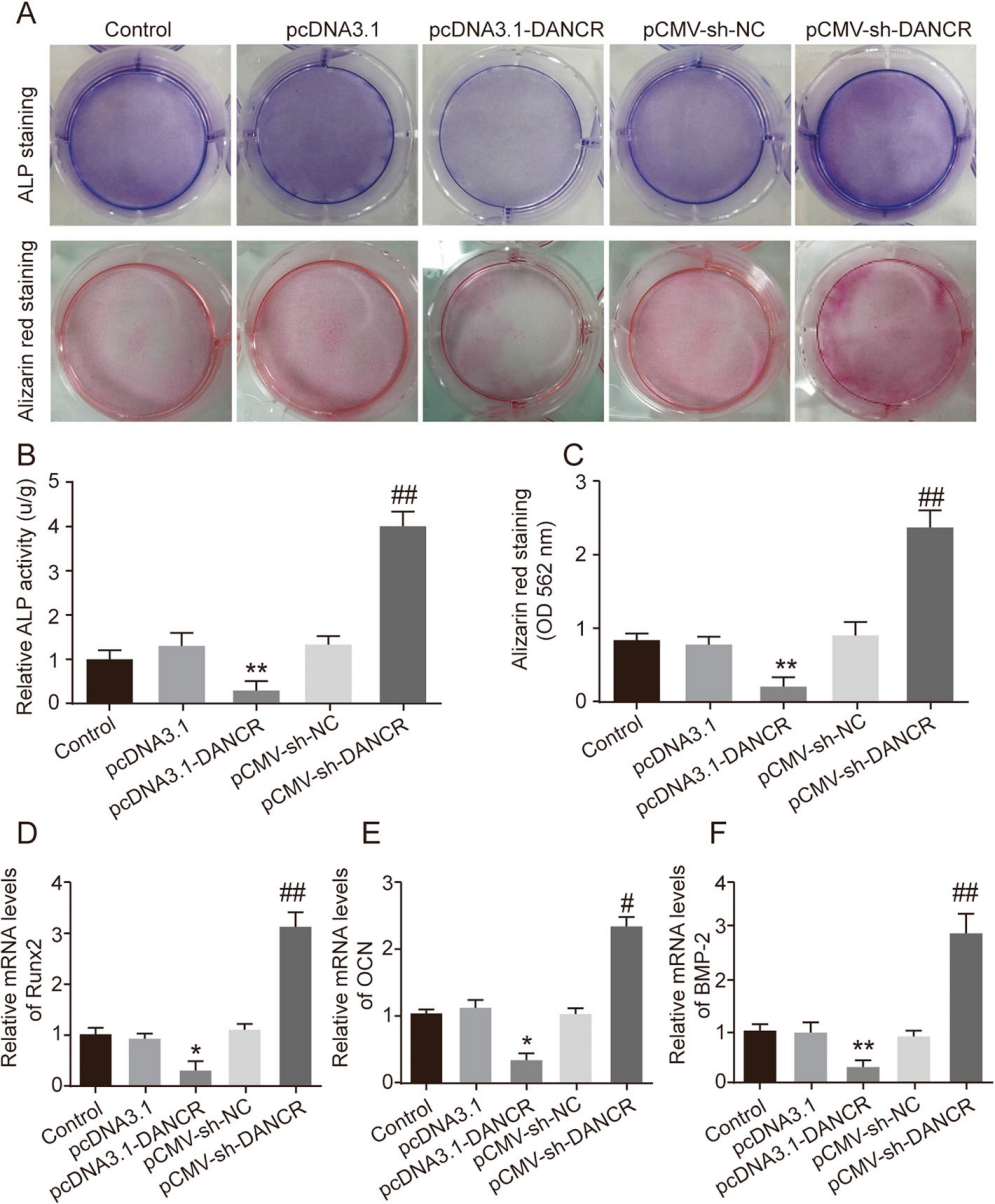
Downregulation of DANCR is positively correlated with the osteogenic differentiation of PDLSCs. Five experiment groups were set up, including the control group, the pcDNA3.1 group, the pcDNA3.1-DANCR group, pCMV-sh-NC and the pCMV-sh-DANCR group. All the experiment groups were grown in an osteogenesis-inducing medium. **a** ALP staining and Alizarin Red staining were performed at the day 7 and 21 after the osteogenic induction on all five experiment groups. Scale bar: 200 μm. **b**-**c** Quantification of ALP activity and cellular calcium level in all experiment groups were measured at different time points after the induction. **d**-**f** qRT-PCR analysis of Runx2, OCN, and BMP-2 mRNA levels in different experiment groups 21 days after the osteogenic induction. *P < 0.05, **P < 0.01, compared with the pcDNA3.1 group; # *P* < 0.05, ## *P* < 0.01 compared with the pCMV-sh-NC group

We have previously demonstrated that the level of DANCR was decreased in PDLSC osteogenic differentiation. Based on this finding, we sought to further investigate the effects of DANCR in the osteoblastic differentiation of PDLSCs. As showed in Fig. 3a and b, both ALP staining and ALP activity analysis suggested that a significant increase in ALP expression in the pCMV-sh-DANCR group compared to the other four groups. However, ALP levels were significantly decreased in pcDNA3.1-DANCR group. Similarly, Alizarin Red staining and the quantification of calcium levels also found calcium deposition in the knockdown of DANCR were markedly increased (Fig. 3a and c). These results were further evidenced by the qRT-PCR analysis of Runx2, OCN and BMP-2. The expressions of Runx2, OCN and BMP-2 were notably decreased in pcDNA3.1-DANCR transfected PDLSCs while significantly increased in pCMV-sh-DANCR group (Fig. 3d-f). Altogether, these data suggested that down-regulating the expression of DANCR promoted the osteogenic differentiation of PDLSCs.

## Discussion

In this study, we aimed at finding the role of lncRNA DANCR in the osteogenic differentiation after transfection of PDLSCs. MTT assay was performed to measure the cell proliferation, and the results indicated that cell proliferation was suppressed in pCMV-sh-DANCR group and was enhanced in pcDNA3.1-DANCR group. And the result of qRT-PCR showed that the osteogenic differentiation marker genes, including Runx2, OCN, and BMP-2, were significantly increased in pCMV-sh-DANCR group. We performed ALP activity and alizarin red staining [15] assays to measure cell osteogenic differentiation, and the results showed that both ALP and alizarin red staining levels were higher in pCMV-sh-DANCR group. All these results suggested that downregulation of lncRNA DANCR promotes osteogenic differentiation of PDLSCs.

Recently, many studies have focused on the functional deficiency of stem cells in several disease conditions. PDLSCs are mesenchymal origin that can differentiate into cementum-forming cells and osteoblasts and thus have been regarded as the most important seed cells for periodontal tissue regeneration [16].Through regulating gene expression by a variety of mechanisms, such as transcription, post-transcriptional processing, chromatin modification and the regulation of protein function, lncRNAs are involved in multiple processes of physiological process including stem cell maintenance and differentiation [17]. Although lncRNAs has been reported as key factors in many organs and biological pathways, their regulation and translation ways in PDLSCs’ osteogenic differentiation have not been fully understood [2]. LncRNA DANCR has many physiological functions, for example, lncRNA DANCR was reported to enhance invasion activity of prostate cancer cells through downregulating the expression of TIMP2/3 [18]. But the functional role of lncRNA DANCR in cell osteogenic differentiation especially in PDLSCs remains largely unclear. Our result revels that lncRNA DANCR has the function of inhibiting cell osteogenic differentiation and promoting proliferation in PDLSCs.

Besides, there are some reports similar to our study. Zhang et al. found that lncRNA DANCR was downregulatedin human bone marrow-derived mesenchymal stem cells (HBMSCs) during osteogenic differentiation, and transfection with si-DANCR significantly enhanced the cell viability and promoted osteogenic differentiation of HBMSCs [9]. And Jia et al. also demonstrated that down-regulated lncRNA DANCR promotes osteogenic differentiation of PDLSCs [19]. LncRNA DANCR could inhibit osteogenic differentiation by regulating fork box transcription factor 1 (FOXO1) expression [20].

Moreover, the molecular mechanism of this differentiation potency is poorly understood. In the study of QU et al., pathway analysis suggested that several kinds of signaling pathways were involved in the osteogenic differentiation process in hPDLSCs, including TGF-beta signaling pathway, Toll-like receptor signaling pathway, MAPK signaling pathway and osteoclast differentiation, etc. [21]. And the mechanism of DANCR on osteoclast differentiation needs further investigation.

At present, lncRNA research has been widely used in the study of osteogenic differentiation, Yang et al. found that lncRNA Reg1cp abnormal mutation can lead to human high bone mass syndrome [22]. The role of lncRNA in osteogenic differentiation and its mechanism have also been reported in large numbers [23–25]. Although this study found that LncRNA DANCR can inhibit osteogenic differentiation and provide a basis for future targeted therapies, a large number of clinical trials and epidemiological investigations are needed to further verify the possibility of implementation. This is also the clinical application of this study. Limitations. However, with the deepening of the mechanism research, we believe that this research can provide experimental basis for future in-depth research.

## Conclusions

Down-regulated of lncRNA DANCR promotes osteogenic differentiation of PDLSCs, which maybe a novel therapeutic strategy that can be used for the dental tissues.

## Methods

### Sample collection and cells culture

The periodontal ligament (PDL) was collected from patients in the Shanghai East Hospital, Shanghai Jiao Tong University School of Medicine. The first or the second premolar teeth were respectively collected from six healthy patients ranging from 18 to 25 years old for orthodontic purposes. This study was approved by the ethics committee of Shanghai East Hospital Affiliated to Tongji University. Written informed consent was obtained from all participants at the beginning of the study.. All experiments were performed in accordance with approved guidelines and regulations from Shanghai East Hospital Affiliated to Tongji University of Medicine Ethics Committee. In this study, the experimental data analysts applied the blind method for data analysis and statistics to avoid deviations in the data processing process.

The extracted teeth were cultured in 90% Dulbecco’s Modified Eagle Medium (DMEM) with 100 u/ml penicillin, 100 μg/ml streptomycin, and 10% fetal bovine serum (FBS). The PDL tissue was obtained by scraping the root surface of teeth after washing 6–8 times with PBS (containing penicillin/streptomycin). The centrifugation of 5 min in 1000 r/min was performed for PDL tissue which was cut into small pieces of 1 mm^3^. Then, the PDL tissue was digested in 3 mg/ml collagenase I (Roche, USA) and dispersed for 45 min at 37 °C. Then, added an equal volume of medium to terminate the digestion. The solution was centrifuge d at 1000 r/min for 5 min, and the supernatant was discarded. Next, the tissues were resuspended in 1 ml of medium and inoculated into 6-well plates and cultured in the environment of 37 °C and 5% CO_2_. After every 2 or 3 days, the medium would be changed until the cells appeared from the edge of the tissue after 3–8 days. Then the cells were cultured as Zhang et al. reported and four generations of poly-clonal PDLSCs were used in this study [2].

### Induction of osteogenic differentiation in PDLSCs

PDLSCs were transferred into a 6 well plate, and per well has the density of 1 × 10^5^ cells. Osteogenic differentiation induction medium (50 mg/mL of ascorbic acid (Sigma, St. Louis, MO, USA), 10 mmol/L of beta-glycerophosphate (BGP, Sigma, St. Louis, MO, USA) and 10 ng/mL of dexamethasone diluted in 10% FBSa-MEM) was given when cells reached 80% confluence. After every 3 days, the conditioned medium was changed. Cells cultured with standard culturing medium were designated as control group. After 21 days of culturing, the differentiated PDLSCs were formed.

### Microarray analysis

The differential expressions of lncRNAs were identified by microarray between undifferentiated and differentiated PDLSCs. A total of six samples (three undifferentiated PDLSCs and three differentiated PDLSCs respectively from six individuals), which each contained about 1 × 10^6^ cells, were prepared, and TRIzol reagent (Invitrogen, Carlsbad, CA, USA) was used to isolate total RNA. And the RNA was amplified and transcribed into fluorescent cDNA. Human LncRNA Array was used to hybridize with labeled samples. Differentially expressed lncRNAs were identified through Volcano Plot filtering with the threshold of fold change > 2 and *P* < 0.05.

### Quantitative real-time polymerase chain reaction (qRT-PCR)

For the extraction of total RNA, TRIzol reagent (Invitrogen, Carlsbad, CA, USA) was used. For quantitative analysis of RNA expression, 1 μg of total RNA was used to synthesize complementary DNA (cDNA) using PrimeScript RT reagent kit (TaKaRa), and the corresponding cDNA was used for quantitative PCR using SYBR green Master MIX (Applied Biosystem) and the ABI 7300 Real-Time PCR System (Applied Biosystems, Foster City, CA, USA). The PCR program, comprising 1 cycle of 10 min at 95 °C, 40 cycles of 30 s at 95 °C, 30 s at 56 °C, and 1 min at 72 °C, and 1 cycle of 7 min at 72 °C. For the endogenous control, GAPDH was used. The primers were shown in Additional file 1: Table S1. The triple analysis was performed for each sample. Relative gene expression was calculated using the 2^-ΔΔCt^ method.

### Plasmid construction and cell transfection

The pcDNA3.1-DANCR and pCMV-sh-DANCR, as well as negative control plasmid were purchased in GenePharma (Shanghai, China). And a total of 3 × 10^5^ cells were transferred into 6-well plates, after 24 h, the negative control plasmid or pcDNA3.1-DANCR or pCMV-sh-DANCR were stably transfected into PDLSCs using Lipofectamine 2000 (Invitrogen, Canada).

### MTT assay

The effect of lncRNA DANCR on cell proliferation was analyzed using an MTT assay. After 24 h of transfection in 100 mm^2^ culture dish, PDLSCs were digested and seeded in 96-well culture plate for another 0, 24, 48, 72 h. Then MTT solution was added and performed as Sun et al. reported [26].

### Alkaline phosphatase (ALP) activity and ALP staining assay

PDLSCs were separately transferred into 96-well plates, and per well has a density of 1 × 10^5^ cells. After 21 days of induction, ALP activity was measured using the ALP assay kit (Jiancheng Technology Co., Ltd., Nanjing, China). Cellular ALP was visualized by using Alkaline Phosphatase Color Development Kit (Beyotime, Shanghai, China) and the methodology is the same as the manufacturer’s protocol.

### Alizarin red staining and quantification

PDLSCs were separately transferred into 24-well plates, and per well has a density of 1 × 10^5^ cells. After transfection, the cells were then cultured for additional 21 days. We changed the induction medium every 3 days. Finally, the 30 min of alizarin red staining measurement was performed by using Alizarin Red S (0.2%, Solarbio, Beijing, China) at room temperature(24–26 °C). After washing in PBS, the cells were observed using an inverted microscope. To measure the concentration of calcium deposits, the Alizarin Red dye in the PDLSCs was extracted with 400 μl of 10% (w/v) cetylpyridinium chloride in 10 mM sodium phosphate solution for 10 min, and then quantified on a UV-Vis spectrometer at 562 nm.

### Statistical analysis

All the statistical analyses were performed by GraphPad Prism (vesion6.0) software. The form of means ± standard deviation (SD) was used for the whole results. Student t-test (unpaired t-test) was used to compare means between differentiated and undifferentiated groups. Differences among the three groups were analyzed via one-way analysis of variance (ANOVA), Tukey’s test was performed in all pairwise comparisons. *P* values < 0.05 were considered as statistically significant.

## Supplementary information

**Additional file 1: Table S1**: The primers sequence of qRT–PCR

## Data Availability

The datasets used and analyzed during the current study are available from the corresponding author on reasonable request.

## Abbreviations

ALP: Alkaline phosphatase
ANOVA: Analysis of variance
ARS: Alizarin red staining
BCIP: 5-Bromo-4-chloro-3-indolyl phosphate
cDNA: Complementary DNA
DMEM: Dulbecco’s Modified Eagle Medium
FBS: Fetal bovine serum
HBMSCs: Human bone marrow-derived mesenchymal stem cells
hPDLSCs: Human periodontal ligament stem cells
LncRNA DANCR: Long non-coding RNA differentiation antagonizing non-protein coding RNA
lncRNAs: Long non-coding RNAs
NBT: Nitro Blue Tetrazolium
OD: Optical density
PDL: Periodontal ligament
PDLSCs: Periodontal ligament stem cells
qRT-PCR: Quantitative real-time polymerase chain reaction
SD: Standard deviation
