# Supplementary figures and images for: Downregulation of lncRNA DANCR promotes osteogenic differentiation of periodontal ligament stem cells
Source: BMC Dev Biol. 2020 Jan 14;20:2. doi: 10.1186/s12861-019-0206-8 (PMC6958786; doi:10.1186/s12861-019-0206-8)

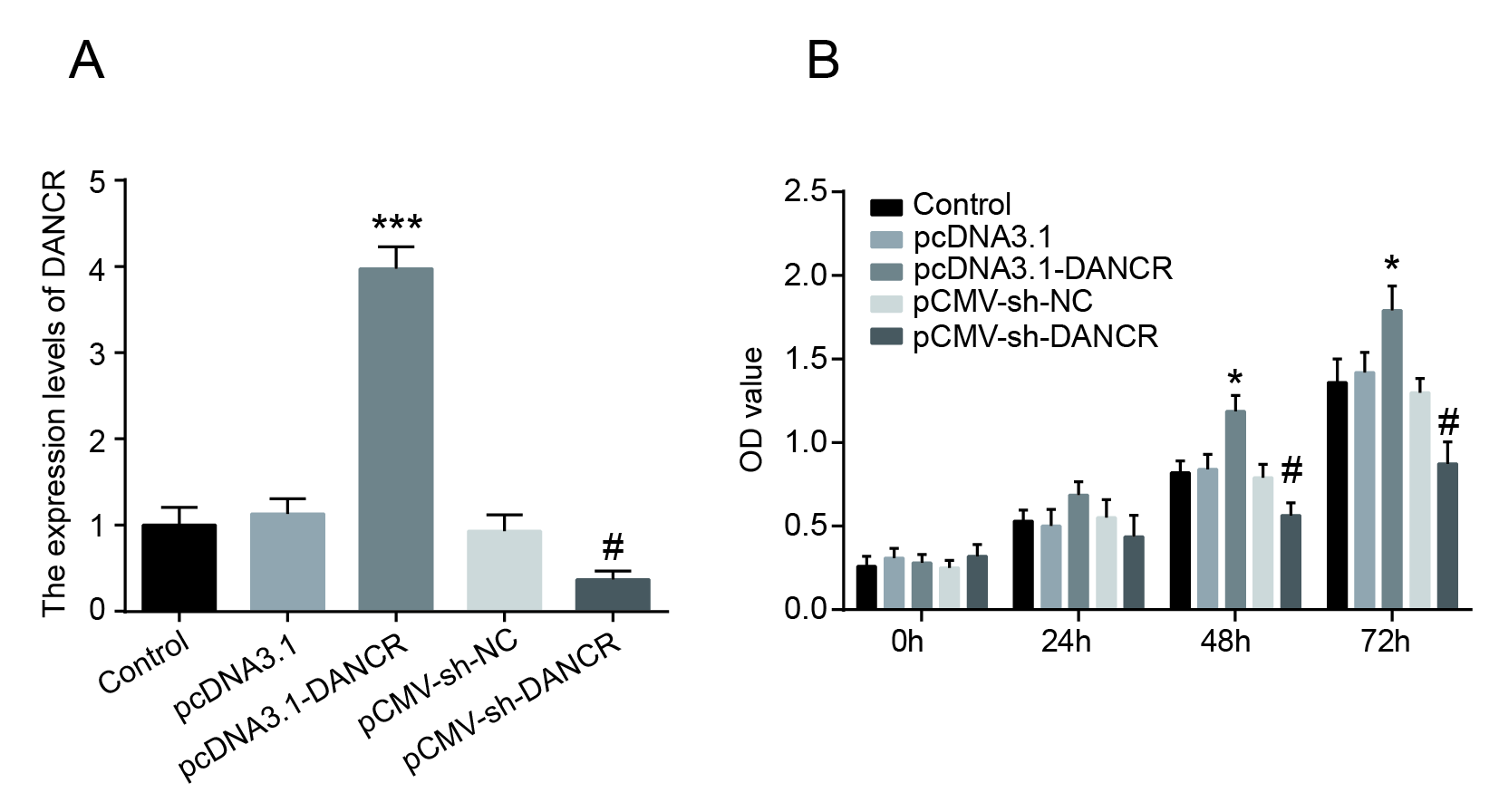

Supplement: Supplementary file 1 — Additional file 1: Table S1: The primers sequence of qRT–PCR [file 12861_2019_206_MOESM1_ESM.tif]
